# A new and accurate qPCR protocol to detect plant pathogenic bacteria of the genus ‘*Candidatus* Liberibacter’ in plants and insects

**DOI:** 10.1038/s41598-023-30345-0

**Published:** 2023-02-27

**Authors:** María Quintana-González de Chaves, Félix Morán, Silvia Barbé, Edson Bertolini, Felipe Siverio de la Rosa, Ester Marco-Noales

**Affiliations:** 1 Unidad de Protección Vegetal, Instituto Canario de Investigaciones Agrarias (ICIA), 38270 Tenerife, Spain; 2 Unidad de Bacteriología, Centro de Protección Vegetal y Biotecnología. Instituto Vaslenciano de Investigaciones Agrarias (IVIA), 46113 Valencia, Spain; 3 Department of Plant Health, Faculty of Agronomys, Federal University of Rio Grande do Sul (UFRGS), Porto Alegre, 91540-000 Brazil

**Keywords:** Pathogens, PCR-based techniques

## Abstract

Four pathogenic bacterial species of the genus ‘*Candidatus* Liberibacter’, transmitted by psyllid vectors, have been associated with serious diseases affecting economically important crops of Rutaceae, Apiaceae and Solanaceae families. The most severe disease of citrus plants, huanglongbing (HLB), is associated with ‘*Ca*. Liberibacter asiaticus’ (CaLas), ‘*Ca*. Liberibacter americanus’ (CaLam) and ‘*Ca*. Liberibacter africanus’ (CaLaf), while ‘*Ca*. Liberibacter solanacearum’ (CaLsol) is associated with zebra chip disease in potatoes and vegetative disorders in apiaceous plants. Since these bacteria remain non-culturable and their symptoms are non-specific, their detection and identification are done by molecular methods, mainly based on PCR protocols. In this study, a new quantitative real-time PCR protocol based on TaqMan probe, which can also be performed in a conventional PCR version, has been developed to detect the four known phytopathogenic species of the genus *Liberibacter*. The new protocol has been validated according to European Plant Protection Organization (EPPO) guidelines and is able to detect CaLas, CaLam, CaLaf and CaLsol in both plants and vectors, not only using purified DNA but also using crude extracts of potato and citrus or psyllids. A comparative analysis with other previously described qPCR protocols revealed that this new one developed in this study is more specific and equally or more sensitive. Thus, other genus-specific qPCR protocols have important drawbacks regarding the lack of specificity, while with the new protocol there was no cross-reactions in 250 samples from 24 different plant and insect species from eight different geographical origins. Therefore, it can be used as a rapid and time-saving screening test, as it allows simultaneous detection of all plant pathogenic species of ‘*Ca.* Liberibacter’ in a one-step assay.

## Introduction

The genus *Liberibacter* comprises Gram-negative bacteria belonging to the Rhizobiaceae family (Class: Alphaproteobacteria, order: Rhizobiales)^1,2^, whose niche is confined to the phloem of host plants and haemolymph and salivary glands of insect vectors that can transmit them. The main vectors of these bacteria are phloem sap-feeding psyllids species (Hemiptera: Psylloidea), in which they multiply in a persistent, circulative form^3–7^. Most of the species of this genus are associated with important plant diseases such as huanglongbing (HLB), the most devastating citrus disease worldwide, the zebra chip of Solanaceae and vegetative disorders of Apiaceae. A remarkable aspect about these bacteria is the fact that they have not been cultured on laboratory media so far^8,9^. Indeed, only one species in the genus, *Liberibacter crescens*, that is non-pathogenic, has been cultivated^10^. The species '*Candidatus* Liberibacter asiaticus' (CaLas), '*Candidatus* Liberibacter africanus' (CaLaf)^1^ and '*Candidatus* Liberibacter americanus' (CaLam)^11^ are associated to HLB. Almost all commercial citrus species and cultivars, regardless of rootstocks, are susceptible to this disease^3,12^.

Typical symptoms of HLB are rapid tree decline, including yellow shoots with blotchy mottled leaves, lopsided fruits with color inversion, aborted seeds, leaf and fruit drop and shoot dieback; the disease causes tree death within a few years^3,13^, producing billions of dollars of economic losses worldwide annually^14^.

HLB is distributed by Asia, America, Oceania and Africa^15^ and it is transmitted mainly by *Diaphorina citri* (Hemiptera: Liviidae) in America and Asia^11,16,17^ and *Trioza erytreae* (Hemiptera: Triozidae) in North Africa^7,18^. Europe is free of the disease, but the vector *T. erytreae* is already present in Spain and Portugal^19,20^. Very recently, other country of the Mediterranean basin, Israel, has reported the presence of *D. citri* in its territory^21^.

The species '*Candidatus* Liberibacter solanacearum' (CaLsol)^22^ is associated with diseases or vegetative disorders in strategic crops, such as potato, tomato, pepper, celery, parsnip and carrot^23–26^. The symptoms are dependent of the host, but the most characteristics are leaf chlorosis and discoloration, wilting, twisted stems, swollen nodes and aerial tubers^7,27,28^, abnormal production of leaves and root sprouts, size reduction and other vegetative disorders^25,29–31^. In short, the commercial quality of the products is devalued, and the economic consequences are important^32^. CaLsol is distributed by America, Europe, North Africa, Israel and New Zealand. The vectors of CaLsol are *Bactericera cockerelli* in America, *Trioza apicalis* in North Europe^33^ and *Bactericera trigonica* in South Europe^30,31^ and North Africa^34^.

There are no curative treatments for diseases caused by '*Ca*. Liberibacter' species (CaLspp). Early and specific detection is essential to prevent the entry of these pathogens into a disease-free zone and limit the dispersion in infected zones. The European and Mediterranean Plant Protection Organization (EPPO) have established standard diagnostic protocols (PM 7) for the molecular detection of HLB associated bacteria^35,36^ and CaLsol^37^. In both cases, real-time PCR methods are recommended for the analysis of vectors and symptomatic and asymptomatic plants^35–37^. These methods are more sensitive, specific, and faster than conventional PCR protocols previously described^1,22,38–41^, and the results are more reproducible^42,43^. In the recently revised PM 7/121 (2) diagnostic protocol for HLB^36^, two screening tests are recommended: the real-time PCR adapted from Li et al.^44^, and a conventional duplex PCR adapted from Teixeira et al.^11^ and Hocquellet et al.^45^, whereas '*Candidatus* Liberibacter' spp. universal real-time PCR by Bertolini et al.^46^ has been removed as it produces false-positive results^36^. However, recent works have demonstrated that both real-time PCRs by Bertolini et al.^46^ and Li et al.^44^, although with a good sensitivity, produced undesired amplifications in environmental samples due to their highly conserved nature of the 16S rRNA gene used as target^47^. Other universal real-time PCR protocols, such as Ananthakrishnan et al.^48^, based on other genes, have been described but not validated according to EPPO requirements^49^. The removal of a universal real-time PCR from diagnostic protocols, such as that of Bertolini et al.^46^, implies the loss of a rapid screening test (RST) that detect all '*Candidatus* Liberibacter' species, including CaLsol, and does not require DNA purification. Therefore, in the present study, our aim was to develop a new highly specific, sensitive and validated qPCR protocol for accurate detection of *'Ca*. Liberibacter' pathogenic species. This protocol could be used as a new genus specific RST tool, suitable for a first screening, as it allows simultaneous detection of all phytopathogenic species of ‘*Ca.* Liberibacter’ in a single step assay. Moreover, it could be used for plant and insect vector sampling with or without DNA extraction, reducing cost and time.

## Results

### Primer and probe design

Primers and probe sequences were designed on a highly conserved region corresponding to the housekeeping gene of the RNA polymerase beta subunit, *rpoB,* with a single copy gen in the genome. The sequence alignment of *rpoB* gene from the nine whole genomes of CaLspp bacteria used in this study showed an Alignment Identity Percentage (AIP) of 91.2%. Additionally, another alignment of 106 sequences of *rpoB* gene region of CaLspp available in NCBI database was done. A graphical representation of the alignment performed with 64 sequences, including two non-pathogenic CaLspp species (*'Ca*. Liberibacter ctenarytaina' (CaLct) and '*Ca.* Liberibacter brunswickensis' (CaLbru)), is shown in Supplementary Fig. S1. Two primers were designed which amplify a region of 156 bp located in the 5’ end of the *rpoB* gene (RefSeq genomic positions 28,333 to 28,178, accession number NC_012985): CaL_rpoB-F (5’-CCT GYA AAC CYT CAT TAG GAC G-3’) and CaL_rpoB-R (5’-TTG TGT TCA ATG GTC TCG GGC GTG- 3’). Primer pairwise identity in the alignment was 96.9% for CaL_rpoB-F and 98.8% for CaL_rpoB-R. Furthermore, a specific TaqMan probe was designed: CaL_rpoB-P (5’-FAM-AGA TCA GGT ATG TCA ATT ATC TCA GG-ZNA4-BHQ1-3’). Probe pairwise identity in the alignment was 99.9%. In order to improve the affinity to the targets and adjust the melting temperature, a ZNA modification (Zip Nucleic Acids, Metabion, Germany) was included in 3’ end.

### Validation of the new qPCR protocol

#### Analytical specificity

Results of new PCR in conventional version for the detection of CaLspp species (CaLas, CaLam, CaLaf and CaLsol) in different hosts (*Solanum tuberosum*, *Daucus carota*, *Citrus* spp., *Vinca* sp., *Bactericera trigonica*, *Trioza apicalis* and *Diaphorina citri*) from several origins (Brazil, Spain, Costa Rica and Finland) showed amplification of a single fragment with a size of 156 bp, and non-specific amplifications were not observed.

Amplification products from positive samples were sequenced. The identity with CaLspp was confirmed using BLAST. CaLaf, CaLam, CaLas and CaLsol infected samples have an AIP ranged 97.06% to 100% with the sequences GenBank accession numbers: CP004021.1^50^, CP006604.1^51^, CP010804.2^52^, CP002371.1^53^ and EU834131.1^40^ respectively. New obtained partial *rpoB* gene sequences were submitted to GenBank database under the following GenBank accession numbers: OM831078 to OM831100.

The new real-time qPCR protocol designed in this study was able to detect CaLas, CaLaf, CaLam and CaLsol on a wide range of samples from different origins and hosts. Specifically, the new protocol was able to detect CaLas in 35 citrus samples from Brazil and Costa Rica, and in 10 samples of the vector *D. citri*. Also was able to detect CaLam in 11 citrus samples from Brazil and from the plant collection of National Research Institute for Agriculture, Food and the Environment (INRAE), and in eight samples of *D. citri*. CaLaf was detected in one plant sample (*Vinca* sp.) from the plant collection of INRAE. CaLsol was detected in 39 *Daucus carota* (including seeds), *S. tuberosum* and *Cuscuta campestris* samples from Spain and Finland; and in 14 samples of the insect vectors *T. apicalis* and *B. trigonica*.

The positive samples were confirmed by previously validated qPCR protocols^25,44,54^. All of them were positive by new protocol, with C_q_ $\left( {{\overline{\text{X}}}_{{{\text{Cq}}}} \pm {\text{SE}}} \right)$ which ranged from 23.0 ± 2.9 to 33.2 ± 4.2 (data not shown). No amplification was detected in the 105 non-target plant and insect samples analyzed, including the strain BT-1 of *L. crescens* and those samples that gave undesired amplifications with other PCR protocols, as described in Morán et al.^47^.

#### Limit of detection (LOD) and standard curve

**Figure 1:**
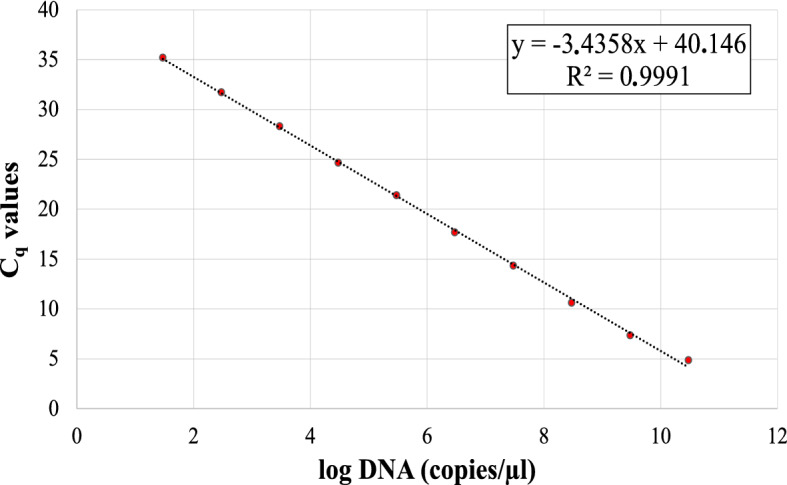
Standard curve for the absolute quantification of target DNA by the new real-time qPCR protocol designed. Quantification cycle values (C_q_) were obtained with the mean of three replicates of ten-fold serial dilutions of target synthetic dsDNA gBlock (from 6·10^10^ to 6 copies µl^−1^).

Absolute quantification by the qPCR protocol designed in this study was performed analyzing known amounts of dsDNA target using serial dilutions of gBlocks (Integrated DNA Technologies, Inc., USA). The results of the linear regression analysis were used to calculate the equation of the standard curve, as the average of the three replicates examined, obtaining a slope of -3.4358 and a correlation coefficient (R^2^) of 0.9991 (Fig. 1), with an efficiency of 95.5%. The LOD, corresponding to the lowest amplified concentration, was 6 copies µl^−1^.

#### Analytical sensitivity

Total DNA purified from selected CaLas- or CaLsol-infected samples of *C. sinensis* (CC140, CC116 and CC114) and *S. tuberosum* (PA), respectively, were quantified by the new real-time qPCR protocol. Results showed that the samples contained 299.8 (CC140), 178.5 (CC116), 149.0 (CC114) and 275.7 (PA) copies·µl^−1^ of target bacteria. These samples were used to prepare spiked crude extracts of *C. lemon*, *C. reticulata* and *S. tuberosum.*

The results of analytical sensitivity of the new qPCR designed are shown in Table 1. CaLas was detected in all direct samples of citrus extract, except for the most concentrated one (1/2). CaLsol was detected in all potato extract samples. The average of C_q_ values $\left( {{\overline{\text{X}}}_{{{\text{Cq}}}} \pm {\text{SE}}} \right)$ obtained at the limit of detection in *C. lemon*, *C. reticulata* and *S*. *tuberosum* spiked crude extracts were 37.2 ± 0.2, 34.8 ± 0.0 and 35.1 ± 0.1, corresponding to 4.5, 3.7 and 6.9 copies·µl^−1^, respectively (Table 1). CaLsol was detected with C_q_ values ranging between 30.4 ± 0.2 and 35 ± 0.1 (Table 1).

**Table 1:** Analytical sensitivity of the new qPCR protocol.

Spiked samples	Dilutions of spiked samples	Bacterial concentration (DNA copies µl^−1^)	C_q_ values of new qPCR without DNA purification
CaLas-infected CC116 sample spiked in *C. lemon* extract	1/2	89.3	–
	1/5	35.7	31.4 ± 0.1
	1/10	17.9	32.9 ± 0.2
	1/15	11.9	34.2 ± 0.4
	1/20	8.9	34.5 ± 0.4
	1/40	4.5	37.2 ± 0.2
CaLas-infected CC114 sample spiked in *C. reticulata* extract	1/2	74.5	–
	1/5	29.8	31.2 ± 0.2
	1/10	14.9	33.3 ± 0.3
	1/15	9.9	33.0 ± 0.0
	1/20	7.4	33.2 ± 0.3
	1/40	3.7	34.8 ± 0.0
CaLsol-infected PA sample spiked in *S. tuberosum* extract	1/2	137.8	31.0 ± 0.5
	1/5	55.1	32.1 ± 0.3
	1/10	27.6	33.0 ± 0.3
	1/15	18.4	33.4 ± 0.3
	1/20	13.8	34.6 ± 0.2
	1/40	6.9	35.1 ± 0.1

Average values $\left( {{\overline{\text{X}}}_{{{\text{Cq}}}} + {\text{SE}}} \right)$ shown come from triplicate PCR reactions from each template. Spiked samples were prepared by serially diluting CaLas-infected *C. lemon* and *C. reticulata* crude extracts or CaLsol-infected *S. tuberosum* crude extract in respective non-infected crude extracts (v/v). The number of DNA copies per microliter was calculated by the new qPCR method from DNA purification.

#### Repeatability and reproducibility

Both parameters, repeatability and reproducibility, were evaluated in the context of a Test Performing Study (TPS 2020 HLB, data unpublished) organized by the European Union Reference Laboratory (EURL) for Bacteriology in the field of Plant Health. Evaluation was performed by six different laboratories, which analyzed 20 DNA samples from CaLas, CaLam, CaLsol and *X. citri* pv. *citri* infected and healthy plants with different protocols. The participating laboratories had sufficient technical expertise for the diagnostic tests. Results showed repeatability and reproducibility of 100% for the new real-time qPCR protocol designed, obtaining agreement on all samples from all TPS participants.

None of the six laboratories obtained amplifications from healthy plant samples, while for ‘*Ca.* Liberibacter’ spp. infected plants they obtained mean C_q_ values and standard error ranging from 21.84 ± 7 to 32.81 ± 1.20. The coefficient of variation obtained was less than 0.06 in all samples (Table 2).

**Table 2:** Results obtained in the Test Performing Study (TPS 2020 HLB) organized by the European Union Reference Laboratory Plant Health for pests of plants on bacteria, showing the average C_q_ values, the standard error, the coefficient of variation and agreements obtained by six laboratories participants.

‘*Ca*. Liberibacter spp’ detected	C_q_ average(± SE)	CV	Agreements (%)
CaLas	21.84 ± 7.00	0.32	100%
CaLam	25.15 ± 1.04	0.04	100%
CaLas	26.03 ± 0.97	0.04	100%
CaLas	25.89 ± 0.99	0.04	100%
CaLas	29.40 ± 0.96	0.03	100%
CaLas	26.09 ± 0.66	0.03	100%
CaLas	28.74 ± 1.32	0.05	100%
CaLas	32.81 ± 1.20	0.04	100%
CaLas	23.87 ± 0.90	0.04	100%
CaLas	27.21 ± 1.16	0.04	100%
CaLas	24.03 ± 0.77	0.03	100%
CaLas	27.50 ± 0.78	0.03	100%
No-CaLspp	0	0	100%
No-CaLspp	0	0	100%
No-CaLspp	0	0	100%
No-CaLspp	0	0	100%
CaLsol	24.02 ± 1.15	0.05	100%
CaLsol	28.07 ± 0.90	0.03	100%
CaLsol	27.86 ± 0.83	0.03	100%
Xcc PD990	0	0	100%
Xcc PD990	0	0	100%

#### Comparison of the new qPCR real-time with other PCR protocols and evaluation in random sampling survey

**Figure 2:**
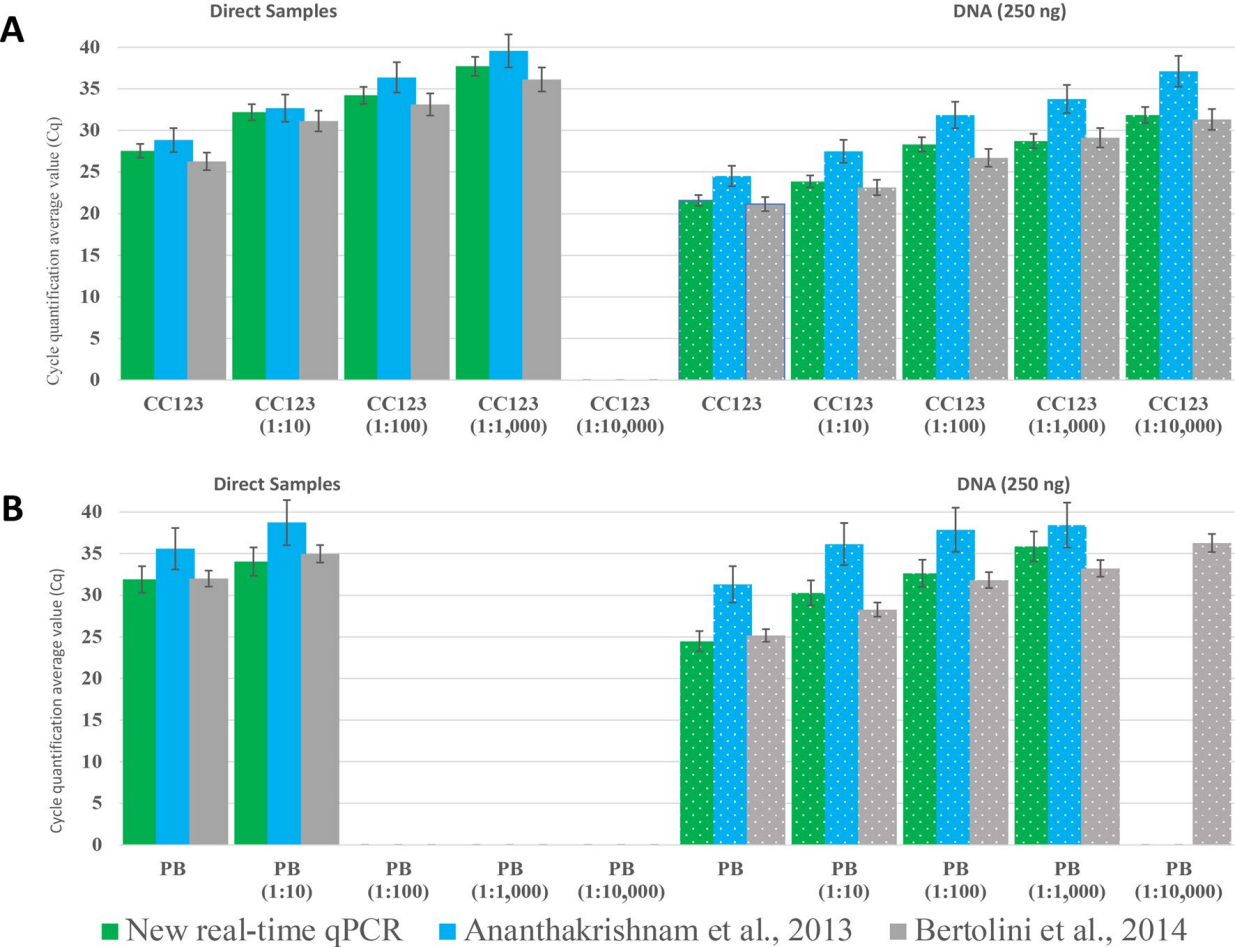
Relative sensitivity (average of C_q_ values) of the new real-time qPCR protocol designed in this study, performed with both direct samples (solid texture) and DNA purifications (dotted texture), in comparison with another two real-time PCR protocols described by Ananthakrishnan et al.^48^ and Bertolini et al.^46^. (**A**) CaLas-infected *Citrus sinensis* cv. Valencia sample (CC123) diluted in healthy plant material. (**B**) CaLsol-infected *Solanum tuberosum* sample (PB) diluted in healthy plant material. Bars represent standard deviations.

The new real-time qPCR designed for the detection of all ‘*Ca*. Liberibacter’ pathogenic species was compared with two real-time PCR protocols previously described by Bertolini et al.^46^ and Ananthakrishnan et al.^48^, both based in TaqMan probes. For this comparison, direct samples, DNA purifications from *C. sinensis* cv. Valencia infected with CaLas (sample CC123) and *S. tuberosum* infected with CaLsol (sample PB) diluted in healthy plant material were analyzed (Fig. 2).

Comparative results of relative sensitivity showed that, in both *C. sinensis* and *S. tuberosum* samples, the mean C_q_ values obtained in all dilutions tested were higher in the direct samples than in the DNA purifications. In the case of direct *C. sinensis* samples (Fig. 2A left), the new real-time qPCR revealed mean C_q_ values ranging from 27.5 ± 0.3 to 37.7 ± 0.5, similar to the values obtained by Bertolini et al.^46^ (26.2 ± 0.4 and 35.1 ± 0.5) and earlier than those obtained using the protocol described by Ananthakrishnan et al.^48^ (28.8 ± 0.8 and 39.5 ± 1.1). For direct *S. tuberosum* samples (Fig. 2B left), the values obtained with the new real-time qPCR protocol ranged from 31.9 ± 0.6 to 34 ± 0.4, again similar to those obtained using Bertolini et al.^46^ (from 32 ± 0.4 to 34.9 ± 0.1), and earlier than those obtained using Ananthakrishnan et al.^48^ (from 35.5 ± 0.3 to 38.7 ± 0.5). For purified DNAs, the new real-time PCR protocol showed mean C_q_ values from 21.5 ± 0.5 to 31.8 ± 0.3 in *C. sinensis* (Fig. 2A right), again like those obtained by Bertolini et al.^46^ (from 21.1 ± 0.7 to 31.3 ± 0.6) and better than those obtained by Ananthakrishnan et al.^48^ (from 24.5 ± 0.7 to 37.1 ± 0.4) except for potato sample PB, where only Bertolini was able to detect the target at dilution 1:10,000.

And in the case of purified DNA in *S. tuberosum* (Fig. 2B right), the mean C_q_values of the new qPCR protocol ranged from 31.9 ± 0.7 to 34.0 ± 0.4, those of Bertolini et al.^46^ from 32.0 ± 0.5 to 34.9 ± 0.2 and those of Ananthakrishnam et al.^48^ from 35.5 ± 1.0 to 38.7 ± 0.7.

**Figure 3:**
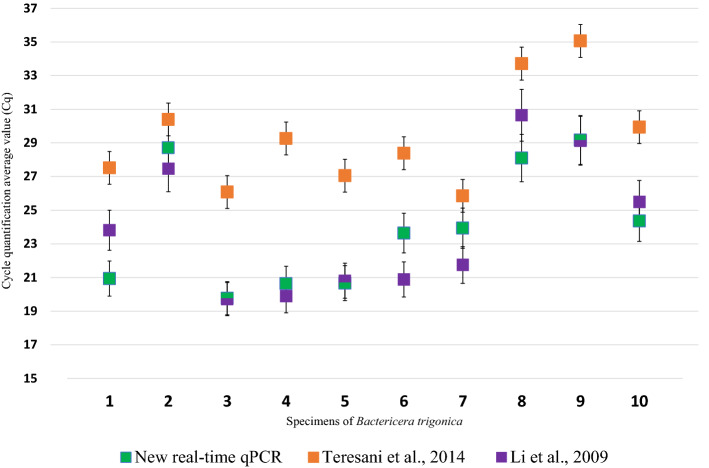
Relative sensitivity (average of C_q_values) of the new real-time qPCR protocol designed in this study, performed with direct samples of ten specimens of *Bactericera trigonica* infected with ‘*Candidatus* Liberibacter solanacearum’, in comparison with real-time PCRsprotocols described by Teresani et al.^25^ and Li et al.^54^.

Relative sensitivity was also comparatively evaluated in direct samples from ten CaLsol-infected *B. trigonica* specimens (Fig. 3). With the new real-time qPCR, mean C_q_values ranging from 19.8 to 29.2 were obtained, with the protocol of Li et al.^54^ from 19.7 to 30.6, and with that of Teresani et al.^25^ later values from 26.1 to 35.1. In four of the ten insect samples, the C_q_values reached by the new real-time protocol were earlier than those with the other two protocols described above, while in the other six samples it was Li et al.^54^ that reached earlier C_q_values (Fig. 3). However, it should be noted that, due to technical limitations, this comparison could only be made with 10 specimens, so the conclusions that can be drawn from these results are preliminary.

## Discussion

A genus-specific tool to detect pathogenic ‘*Ca.* Liberibacter’ bacterial species is essential for the management of important plant diseases, such as HLB, the most devastating disease affecting *Citrus* spp., associated with CaLas, CaLam and CaLaf, and Zebra Chip, an economically important disease of potato associated with CaLsol. Some genus-specific real-time PCR protocols were previously published^46,48,55^; however, several papers have highlighted the high risk of obtaining non-specific amplification in non-target samples^19,43,47^. Accurate early detection is imperative to avoid decisions based on false positive and false negative results, which can have very detrimental consequences for the agriculture and related sectors.

In the present study, we have designed, developed and extensively assessed a new accurate one-step qPCR protocol that could be adopted to facilitate the diagnostic work of the important diseases associated with ‘*Ca.* Liberibacter’ bacterial species.

Moreover, it can detect the target bacteria directly from the plant material without the need for DNA extraction, which reduces the risk of contamination, saves time, decreases the cost per reaction and allows processing a large number of samples. Moreover, the protocol also works in conventional PCR version.

The target region of the new protocol designed is located in the RNA polymerase β subunit gene (*rpoB*), a monocopy gene with a ranging size from 4140 to 4197 bp for the four pathogenic species of CaLspp analyzed in this study. This housekeeping gene is highly conserved, and it is involved in the transcription process and the regulatory pathways that control gene expression in all living organisms^56,57^. In addition, *rpoB* gene has been demonstrated to be useful in providing resolution within groups of closely related bacteria and may refine phylogenetic identification of bacteria^58^. Most of the CaLspp qPCR protocol are targeted on the 16S rRNA gene^46,55^. This gene had been the most used gene for primers development in detection of CaLspp^1,22,25,39–55,59–63^, because it is a highly conserved gene for cell functions^64^ and there are three copies presented in the genome of CaLspp bacteria, which increase the PCR sensitivity^14,65^. However, although the sensitivity of the16S rRNA-based protocols is very high, due to the targeted gene copy number, several papers have reported non-specific amplifications^19,43,47^.

Regarding the sensitivity of the new qPCR protocol, the standard showed a high efficiency (95.5%) with a high correlation coefficient (R^2^ = 0.9991). Additionally, it showed the better LOD, 6 synthetic DNA copies µl^−1^, among the current available protocols, which set their limit in 10–20^48,54,55,63,65^. The efficiency, much higher than, for example, Ananthakrishnan et al.^48^ (E CaLas = 82.3%, E CaLam = 78.2% and E CaLaf = 77.8%), is only surpassed by the protocol designed by Orce et al.^55^ (E = 98.0%), but the advantage of new qPCR method is that it has been tested on many more matrices, and can also be adapted to conventional PCR format.

The specificity of the protocol developed in this study was widely tested for inclusivity, with samples naturally infected with CaLsol or the three bacterial species associated with HLB, and for exclusivity, with non-targeted samples of host plants. This is the first time that a set of universal primers and probe for CaLspp phytopathogenic species detection is tested with almost all its known plant and insect hosts. Orce et al.^55^ tested their new designed qPCR set of primers in symptomatic citrus tissues and some citrus-related pathogens, such as *Xanthomonas citri* subsp. *citri* among others. Ananthakrishnan et al.^48^ tested their primers and probe with CaLsol infected plants, psyllids, HLB-associated species and eight endophytic bacteria: *Paenibacillus glucanolyticus*, *Microbacterium* sp., *Pantoea agglomerans*, *Pseudomonas* sp., *Enterobacter cloacae*, *Rhizobium* sp., *Agrobacterium tumefaciens*, *Sinorhizobium* sp., and the two citrus pathogens *Xanthomonas axonopodis* pv. *citri* and *X. axonopodis* pv. *citri* A^w^; and they did not find unspecific amplifications.

Recently, a paper by Morán et al.^47^ focused on the unspecific amplifications obtained in citrus plant and insect samples, revealing that almost 10% of the samples amplified by Bertolini et al.^46^ were, in fact, non-infected; besides, authors also found cross reactions in samples by using the protocol developed by Li et al.^44^. Some of these samples, which show unspecific amplifications, described by Morán et al.^47^ were analyzed in the present study, and the result by the new qPCR protocol was negative, providing evidence of the high specificity of the new designed primers and probe. After sequencing, Morán et al.^47^ found that unspecific amplifications obtained in the samples, which have also been used in the present study, were due to cross reactions with *Asaia* sp. in *T. dryi*, *Rhizobium* sp. and *Sphingomonas* sp. in *M. koenegii*, *Sphingomonas* sp. and *Phyllobacterium* sp. in *Citrus* spp., and uncultured bacterium and *Sodalis* sp. in *T. erytreae.* All these microorganisms are associated to plants and/or insects, and share habitat with CaLspp^47,66–70^; for this reason, it is important to have specific detection methods for CaLspp that do not amplify other microorganisms that share the same niche, causing unspecific cross-reactions.

Since PCR inhibition due to plant or insect tissues may affect sensitivity, different experiments were carried out in the present study to evaluate the selectivity of the new qPCR method. CaLas was detected in the most spiked dilutions of citrus plants, both in plant extract material and DNA purification, obtaining better results using nucleic acid purifications. This fact suggests that citrus tissues may present PCR inhibitors, which can be easily avoided diluting the sample, as recommended by EPPO for other pathogens, such as *Xylella fastidiosa*^71^. In the case of CaLsol detection, also the new qPCR protocol was able to detect it even when the DNA was not extracted, directly from potato crude extract samples. The new qPCR method reached higher sensitivity in potato DNA purification than in their corresponding direct samples. This fact evidenced the existence of PCR inhibitor components in potato tissue which could affect the reaction efficiency. For this reason, it is advisable to perform DNA purification for the detection of CaLsol in asymptomatic potato tissues infected with low titers of bacteria. And only analyze direct potato samples when they show symptoms^35–37,71^.

All these data suggest that this new method could detect the target organisms directly from plant material without DNA purification with acceptable efficiency (LOD 3.7–6.9 copies µl^−1^ in spiked samples). The use of the method without DNA extraction reduces the risk of contamination, saves time and lowers the cost per sample^46,72^. Therefore, this variable of the described method would be very useful for large scale surveys. Regarding the DNA extractions, it would be useful for more sensitive analysis of plant material, e.g. in disease-free areas and/or asymptomatic samples.

Quantification cycle values (C_q_) were used to relatively compare the sensitivity of the new qPCR protocol with previously designed Ananthakrishnan and Bertolini^46,48^. Firstly, it was studied the sensitivity of the CaLas detection in the citrus samples, obtaining few differences between methods. It should be highlighted that, in most of cases, the new protocol has a sensitivity comparable to other protocols for CaLspp detection, despite the fact that Bertolini et al.^46^ protocol, as discussed above, is based on the 16S rRNA gene, which has three copies in the CaLspp genomes^14,65^, giving it an advantage in sensitivity. Secondly, CaLsol sensitivity was evaluated with potato infected samples, in which Ananthakrishnan method^48^ showed the worst sensitivity in almost all samples tested. Once again, the new protocol was, in most cases, the most sensitive in terms of CaLspp detection. These comparisons provide further data demonstrating the high sensitivity of the new protocol, opening up the possibility of using it as a more accurate RST tool than the current ones.

In conclusion, the new one-step qPCR TaqMan assay is a specific, sensitive and accurate tool, being able to detect a low number of target copies per microliter. The use of this new protocol could improve and reduce the work in plant health laboratories since it allows the screening of the four phytopathogenic bacteria present in this genus and does not present cross reactions or non-specific amplifications with different host plants or insect species.

## Methods

### Plant and insect material

Collections of plants and insects were carried out in accordance with national and international legislation in force since it did not require any special permit because they were in open access areas. Likewise, experimental research on plant and insect material was carried out in accordance with national and international legislation in force, as no special permits were required. A total of 250 samples from eight different origins and 24 different plant and insect species were used for analytical specificity of the qPCR method developed in the present study. For the validation of the method, 144 samples of CaLsol-, CaLas-, CaLam- and CaLaf-infected plants and insects were selected as positive controls: 45 CaLas-infected samples from Brazil and Costa Rica; one CaLaf-infected sample from INRAE collection; 19 CaLam-infected samples from Brazil and INRAE collection; and 79 CaLsol-infected samples from Finland and Spain. A total of non-infected 42 plant (of 15 different species) and 36 insect samples (from three species) were used as negative controls.

Twenty-six plant and insect samples previously reported as undesired amplifications by Moran et al*.*^47^ and the strain BT-1 of *Liberibacter crescens* were included in the validation.

In addition, 48 carrot samples randomly collected in commercial plots from Villena (Alicante, Spain) were also included.

The presence of ‘*Ca.* Liberibacter’ spp. in control samples was confirmed using the real time PCR detection protocols described by Li et al*.*^44^ and Bertolini et al*.*^46^, in the case of HLB associated CaLspp, and Li et al*.*^54^ and Teresani et al*.*^25^, in the case of CaLsol.

### Sample preparation

Plant crude extracts were prepared by crushing leaves, seeds and tuber potato from each plant sample in extraction bags (Bioreba, Switzerland) with 10 mM phosphate buffered saline (PBS) (8.0 g NaCl; 2.9 g Na_2_HPO_4_·12 H_2_O; 0.2 g KH_2_PO_4_; 0.2 g KCl; pH 7.2) at 1/5 (w/v) ratio. DNA was purified from 400 µl of these crude extracts following the CTAB protocol recommended by EPPO^35–37^. Total DNA was quantified by using a NanoDrop ND-100 spectrophotometer (NanoDrop Technologies, USA).

Regarding insect crude extracts, single specimens were squashed on Whatman paper (GE Healthcare, Europe), according to the procedure developed by Bertolini et al*.*^46^. Then, membranes were resuspended with 100 µl of Tween 20 at 0.05%, and the suspension directly analyzed by qPCR.

Both plant and insect direct samples consisted of 1/10 dilutions of crude extracts in PBS. All total purified DNA and direct samples were kept at −20 °C until use.

### Primer and probe design

In order to find the most conserved region for the design of primers and probe, nine whole genome sequences, from species of ‘*Ca.* Liberibacter’ from different hosts and origins, were aligned using the software package MAUVE^73^: CaLsol (GenBank accession number: NC_014774), CaLas (NC_012985, NC_020549, NZ_AP014595, NZ_CP010804, NZ_CP019958 and NZ_CP029348), CaLam (NC_022793) and CaLaf (NZ_CP00402). The best alignment identity percentages (AIP) in the most conserved region were used. To improve the design of the highly conserved region selected, a second alignment with 106 sequences of this region of *Liberibacter* species available in NCBI data bases was performed using Clustal W^74^, implemented in MEGA 7 software^75^.

### Amplification conditions by conventional PCR and quantitative real-time PCR

Conventional PCR amplifications were carried out to test the primers, using 2 U Taq DNA polymerase (Biotools, Spain), 1 µM of each primer, 0.1 mM of each dNTPs, 1.5 mM MgCl_2_ and 5 µl of DNA template (50 ng), in a total reaction volume of 25 µl. Conventional PCR conditions consisted of a first denaturalization step at 94 °C for 3 min, followed by 40 cycles of amplification (94 °C for 30 s, 63 °C for 30 s and 72 °C for 45 s) and a final extension step at 72 °C for 10 min. A Veriti Dx Thermal Cycler (Thermo Fisher Scientific, USA) was used. The amplification products obtained were visualized through 1.5% (w/v) agarose gel electrophoresis in 0.5X TAE buffer (40 mM Tris pH 7.6, 20 mM CH_s_COOH, 1 mM EDTA) with Good View™ staining (SBS Genetech Co., Ltd., China).

Quantitative real-time PCR (qPCR) reaction mix optimized using GoTaq® DNA Probe qPCR Master Mix (Promega Corporation, USA) consisted of 500 nM of each primer (CaL_rpoB-F and CaL_rpoB-R), 80 nM of TaqMan probe (CaL_rpoB-P) and 5 μl of DNA template (50 ng) or crude extract as template in a total volume of 25 µl. To normalize fluorescent signals between wells, 30 µM of CXR reference dye was added to the Master Mix. The conditions of qPCR assay consist of an initial denaturalization step at 95 °C for 10 min, followed by 45 cycles of amplification (95 °C for 15 s and 60 °C for 1 min). A StepOne Plus qPCR thermocycler (Applied Biosystems, USA) and a LightCycler 480 thermocycler (Roche, USA) were used.

Default threshold lines set by the thermocycler software were adjusted slightly above the background noise, according to the manufacturers, to obtain the cycle quantification value (C_q_). All samples were analyzed in triplicate.

A selection of 23 PCR products, obtained from different bacterial hosts originating from Brazil, Costa Rica, Finland, Spain, and USA (Table 3), were purified using mi-PCR Purification Kit (Metabion International AG, Germany), and sequenced through Sanger sequencing methods by Macrogen sequencing service (Macrogen Inc., Spain) in both directions (forward and reverse complimentary DNA strands). Sequences were analyzed using MEGA 7 software^75^.

**Table 3:** Plant and insect samples used in the present study.

CaLspp^1^	Origin	Host	N	Id sample
CaLas	Costa Rica	*Citrus sinensis*	27	CC44, CC46, CC104, CC108, CC114, CC116, CC123**, CC124**, CC140, CC141**, CC144, CC146, CC400, CC409-CC413, CC415-CC420, CC422, CC423, CC1096
CaLas	Brazil	*Citrus sinensis*	18	BCLas11-BCLas21, 4127**, 4224**, 4258**, 4259**, 4327**, 4535**, 4537**
CaLas	Brazil	*Diaphorina citri*	10	BPLas9**-BPLas18
CaLas	EEUU	*D. citri*	3	11,662.7**, 11,662.9**, 11,662.21**
CaLam	Brazil	*D. citri*	8	BPLam1**-BPLam8
CaLam	Brazil	*C. sinensis*	10	BCLam1**-BCLam10
CaLam	INRAE collection	*Citrus* sp.	1	CAM**
CaLaf	INRAE collection	*Catharantus roseus*	1	PAF**
CaLsol	Finland	*Trioza apicalis*	10	T1a, T1b, T2a, T2b, T3a, T3b, T4a**, T4b, T5a, T5b
CaLsol	Finland	*Cuscuta campestris*	8	T5 (3), T6 (3), T8 (4), T9 (D2), T10 (N6), T11 (N6), T12 (4), T13 (4)
CaLsol	Finland	*Daucus carota* (leaves)	7	T2, T14, T18, T19, T3, T5, T6
CaLsol	Spain	*Bactericera trigonica*	30	C:1**-5, W: 1–5; C:1–5; P:1–5; M: 1–10
CaLsol	Spain	*D. carota* (leaf)	20	8E, 4B, 3G**, 8D, 8C, 2D, 9B, 4G, 6F, 8B, 6B, 4C, 1C, 1F, 7E, 6A, 7B, 6G, 1B, 2G
CaLsol	Spain	*D. carota* (seed)	1	sem +
CaLsol	Spain	*Solanum tuberosum*	3	PA**, PB, PC
No-CaLspp	South Africa	Tamarixia dryi*	19	T7, T31, T33, T38, T46, T66, T67, T212, T219, T227, T244, T247, T257, T260, T267, T278, T540, T575, T576
No-CaLspp	Uganda	Murraya koenegii*	2	IVIA 5020, IVIA 5029.2
No-CaLspp	Thailand	*Citrus* sp.*	1	IVIA 5029.1
No-CaLspp	Spain	Trioza erytreae*	2	i273, i800
No-CaLspp	Spain	*Citrus* sp.*	2	P15, P16
No-CaLspp	Spain	*Citrus reticulata*	6	M7-M9, M28-M30
No-CaLspp	Spain	*C. sinensis*	6	N1-N3, N22-N24
No-CaLspp	Spain	*Citrus lemon*	6	L4-L6, L25-L27
No-CaLspp	Spain	*Citrus* × *aurantifolia*	1	LiTF
No-CaLspp	Spain	*T. erytreae*	12	epg1, epg5, epg11, epg2.1, epg2.2, epg2.3, epg3, epg12.3, epg14.4, epg2.2, epg30.3, epg7.1
No-CaLspp	Spain	*B. trigonica*	19	B1**-B9, B1.1-B1.10
No-CaLspp	Spain	*Bactericera nigricornis*	5	Bn1-5
No-CaLspp	Spain	*D. carota* (leaves)	1	Z-
No-CaLspp	Spain	*S. tuberosum*	4	PA1, PA2, PB1, PB2
No-CaLspp	Spain	*Coriandrum sativum*	1	Ci1
No-CaLspp	Spain	*Petroselinum crispum*	1	PE1
No-CaLspp	Spain	*Foeniculum vulgare*	1	Hi1
No-CaLspp	Spain	*Torilis* sp.	1	To1
No-CaLspp	Spain	*Cyclospermum* sp.	1	Cy1
No-CaLspp	Spain	*Ferula linkii*	2	Fe-ant, Fe1
No-CaLspp	Spain	*S. tuberosum*	1	papaA, papaB
No-CaLspp	Spain	*Capsicum annuum*	1	Pi1
No-CaLspp	Spain	*Solanum lycopercisi*	2	To1, To2
No-CaLspp	Finland	*C. campestris*	7	E8, E9, E10, E11, E12, F1, F2
*L. crescens*	Puerto Rico	Pure bacterial culture	1	BT-1^10^

*Samples previously described as undesired amplifications by Morán et al*.*^47^.

**Samples selected for partial Sanger sequencing of the *rpoB* gene.

^1^Detection of CaLspp were done by qPCR following the protocols designed by Li et al*.*^44,54^; Teresani et al*.*^25^; Bertolini et al^46^.

### Validation of the qPCR designed

Validation of the new real-time qPCR protocol developed in this study was performed according to PM 7/98 EPPO guidelines^49^. The following parameters were evaluated: analytical specificity for inclusivity (detection of CaLspp strains from different origins, hosts and genetic diversity) and for exclusivity (negative detection of relevant non-targets that might be present in the same matrix), selectivity (evaluation of different host species), limit of detection (LOD) and standard curve, analytical sensitivity (maximum dilution of target DNA detected) and repeatability and reproducibility (evaluation of the test performance consistency on different replicates by different operators and with different equipments).

### Analytical specificity

It was evaluated for inclusivity and exclusivity with target and non-target samples, respectively. As described above, a total of 250 plant and insect samples from different geographical origins and hosts, including some samples reported previously as undesired amplifications in Morán et al.^47^, were evaluated using three repetitions per sample (Table 3).

### LOD and standard curve

For qPCR standard curve generation, synthetic double stranded DNA commercially named gBlock (Integrated DNA Technologies, USA) with the sequence of a partial region (156 bp) of the *rpoB* gene from CaLsol whole genome (NC_014774), containing the PCR target designed in this study, was used.

### Analytical sensitivity

It was determined testing six serial dilutions (from 1/2 to 1/40), and their corresponding ten-fold dilutions in PBS of spiked crude extract samples of *C. lemon*, *C. reticulata*, and *S. tuberosum*. Thus, non-infected *Citrus* spp. extracts were spiked with DNA isolated from CaLas-infected citrus samples CC116 and CC144 (Table 3); and non-infected *S. tuberosum* extracts were spiked with CaLsol-infected potato crude extract sample PA (Table 3). For all cases, three replicates per sample were used, and the absolute quantification was done as described in the above section (LOD and standard curve). The presence of CaLspp bacteria in tissues was confirmed by qPCR^25,44,46,54^, and quantified by generating standard curves as described above with the new real-time qPCR protocol.

### Repeatability and reproducibility

It was evaluated in the context of a Test Performance Study (TPS) organized in 2020 by the Netherlands Institute for Vectors, Invasive plants and Plant health (NIVIP): TPS for the molecular detection of regulated ‘*Ca.* Liberibacter’ species in *C. sinensis* and *C. reticulata* using conventional and real-time PCR tests (TPS 2020HLB). Eight laboratories from different countries (Italy, Belgium, Slovenia, Netherlands, France, Brazil, Spain, and Japan) participated in the TPS. Twenty samples were tested according to the agreement of qualitative reported results of biological duplicates, i.e. positive or negative, under repeatability and reproducibility conditions, per TPS participant or between TPS participants, respectively, according to EPPO standard PM7/76 (5)^76^.

### Comparison with different real-time PCR protocols

On the one hand, the sensitivity of the new qPCR protocol was compared with two real-time PCR protocols for ‘*Ca* Liberibacter’ spp. detection previously described, based on TaqMan probes, which have as target the 16S rRNA and *rpoB* housekeeping genes (Bertolini et al.^46^ and Ananthakrishnan et al.^48^, respectively). For this comparison, ten-fold serial dilutions (from 10^–1^ to 10^–4^) from the samples CC123 (*C. sinensis* cv. Valencia naturally infected with Calas) and PB (*S. tuberosum*, naturally infected with CaLsol) were prepared in healthy plant material. On the other hand, the sensitivity of the new qPCR protocol was also compared with two real-time PCR protocols for CaLsol specific detection previously described (Li et al.^54^; Teresani et al.^25^) with psyllids. For it, ten psyllid samples of *B. trigonica* naturally infected with CaLsol were used*.* Direct samples and DNA purifications were analyzed with three repetitions by the real-time PCR protocols. Average C_q_ values and standard deviations were used for comparison of the relative sensitivity between the three real-time PCR methods.

## Supplementary Information

Supplementary Figure S1.

## Data Availability

All the datasets and material generated and analyzed during the current study are available from the corresponding author on reasonable request. The sequences datasets generated and/or analyzed during the current study are available in the GenBank repository (OM831078; OM831079; OM831080; OM831081; OM831082; OM831083; OM831084; OM831085; OM831086; OM831087; OM831088; OM831089; OM831090; OM831091; OM831092; OM831093; OM831094; OM831095; OM831096; OM831097; OM831098; OM831099 and OM831100).
