# Supplementary material for: A new and accurate qPCR protocol to detect plant pathogenic bacteria of the genus ‘Candidatus Liberibacter’ in plants and insects
Source: Sci Rep. 2023 Feb 27;13:3338. doi: 10.1038/s41598-023-30345-0 (PMC9971166; doi:10.1038/s41598-023-30345-0)
Supplement: Supplementary file 1 — Supplementary Figure S1. [file 41598_2023_30345_MOESM1_ESM.pdf]

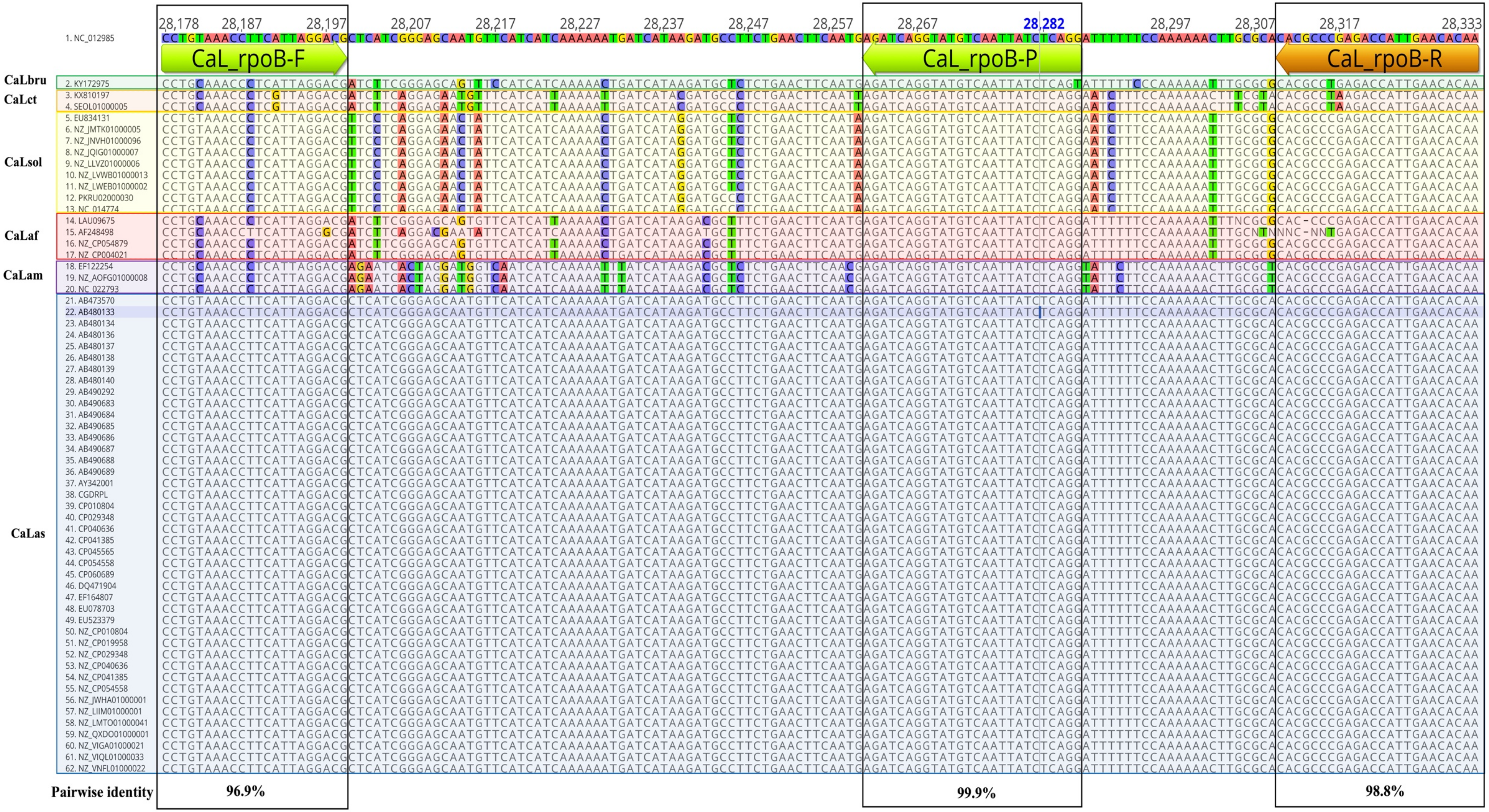

Supplementary figure S1. Nucleotide alignment of 156 nt corresponding to a partial region gene located in the 5' end of the *rpoB* gene (RefSeq genomic positions 28,333 to 28,178 bp, accession number NC_012985) from 64 '*Candidatus* Liberibacter' spp. available in the database. GenBank number from each isolate is indicated. Different species are displayed in colored blocks: '*Candidatus* Liberibacter asiaticus' (CaLas) in blue; '*Candidatus* Liberibacter americanus' (CaLam) in purple; '*Candidatus* Liberibacter africanus' (CaLaf) in red; '*Candidatus* Liberibacter solanacearum' (CaLsol) in yellow; '*Ca.* Liberibacter ctenarytainae' (CaLct) in orange and '*Ca.* Liberibacter brunswickensis' (CaLbru) in green.
